# Comparison of Patient Self-reported Health Status With Clinician-Assigned New York Heart Association Classification

**DOI:** 10.1001/jamanetworkopen.2020.14319

**Published:** 2020-08-28

**Authors:** Andy T. Tran, Paul S. Chan, Phillip G. Jones, John A. Spertus

**Affiliations:** 1 Cardiovascular Research, St Luke’s Mid America Heart Institute, Kansas City, Missouri; 2 Department of Biomedical and Health Informatics, University of Missouri–Kansas City, Kansas City

## Abstract

This cross-sectional study examines clinician-assigned New York Heart Association (NYHA) classification vs patient-reported health status using the Kansas City Cardiomyopathy Questionnaire.

## Introduction

Current clinical trials categorize the severity of heart failure (HF) by the New York Heart Association (NYHA) classification to ensure that enrolled patients have similar disease severity, despite its known limited reproducibility and significant interphysician variability.^1^ Alternatively, the severity of symptoms and functional limitations can be directly elicited from patients using the well-validated Kansas City Cardiomyopathy Questionnaire (KCCQ).^2^ We compared the range of KCCQ scores within NYHA classes in recent clinical studies.

## Methods

### Study Population

This cross-sectional study used 1 prospective cohort study and accessed deidentified data from 2 clinical trials through the National Heart, Lung, and Blood Institute, with institutional review board approval from Saint Luke’s Hospital of Kansas City, Missouri, which granted a waiver of informed consent.

For HF with reduced ejection fraction, we used the KCCQ Interpretability study (KCCQINT),^3^ an observational registry from February 2001 to January 2002, and the HF-ACTION trial,^4^ a trial of exercise training conducted between August 2006 and January 2012. For HF with preserved ejection fraction, we used the TOPCAT trial of spironolactone that enrolled people from August 2006 to January 2012.^5^ Because of known concerns about data validity in TOPCAT, patients from the countries of Russia and Georgia were excluded.

### Statistical Analysis

All analyses were performed separately for each study. The KCCQINT study consisted of all NYHA classifications, HF-ACTION enrolled NYHA classes II to IV, and TOPCAT combined NYHA class I with II (I-II) and III with IV (III-IV). Density plots were constructed to examine the range of KCCQ Overall Summary (KCCQ-os) scores across NYHA classes. We also compared the proportion of patients with a KCCQ-os score greater than or equal to 80, a very high level of functioning, assigned different NYHA classes. Categorical variables are reported as proportions, and continuous variables are reported as mean (SD). All analyses were conducted using R statistical software version 3.6.2 (R Project for Statistical Computing) from September 2019 to May 2020.

## Results

The KCCQINT study had a total of 546 participants (mean [SD] age, 61.0 [13.1] years; 130 women [23.8%]). The HF-ACTION trial had a total of 2129 participants (mean [SD], age 58.6 [12.7] years; 599 women [28.1%]). The TOPCAT trial had 1725 total participants (mean [SD] age, 71.5 [9.7] years; 861 women [49.9%]).

Marked overlaps were noted in KCCQ-os scores by NYHA across all studies (Table and Figure). Participants with a KCCQ-os score greater than or equal to 80, considered good to excellent self-reported health status, were present in 148 of 546 participants (27.1%) in KCCQINT, of whom 39 (26.4%) were assigned to NYHA class I, 81 (54.7%) to class II, 28 (18.9%) to class III, and none to class IV. Among the 31.8% (677 of 2129) in HF-ACTION with good to excellent self-reported health status, 548 (80.9%) were assigned to NYHA class II, 128 (18.9%) to class III, and 1 (0.1%) to class IV. Among the 23.1% (399 of 1725) with good to excellent self-reported health status in TOPCAT, 340 (85.2%) were coded as NYHA class I-II and 59 (14.8%) were coded as class III-IV.

**Table.** NYHA and Baseline Demographic Characteristics by KCCQ-os Scores

Characteristic, NYHA class	KCCQ-os score, Participants, No. (%)				
	0-39	40-59	60-79	80-100	Total
KCCQINT, participants, No.	115	139	143	148	546
Class I	1 (0.9)	6 (4.3)	13 (9.1)	39 (26.4)	59 (10.8)
Class II	23 (20)	49 (35)	74 (51.7)	81 (54.7)	227 (41.6)
Class III	75 (65.2)	77 (55)	51 (35.7)	28 (18.9)	231 (42.3)
Class IV	16 (13.9)	8 (5.7)	5 (3.5)	0 (0)	29 (5.3)
Age, mean (SD), y	58.9 (14.0)	62.0 (13.3)	60.2 (13.0)	62.6 (12.0)	61.0 (13.1)
Female	32 (27.8)	31 (22.1)	37 (25.9)	30 (20.3)	130 (23.8)
White	76 (66.1)	94 (67.1)	98 (68.5)	102 (68.9)	370 (67.8)
HF-ACTION, participants, No.	242	541	669	677	2129
Class II	75 (31)	263 (48.6)	464 (69.4)	548 (80.9)	1350 (63.4)
Class III	159 (65.7)	273 (50.5)	199 (29.7)	128 (18.9)	759 (35.7)
Class IV	8 (3.3)	5 (0.9)	6 (0.9)	1 (0.1)	20 (0.9)
Age, mean (SD), y	54.2 (12.3)	58.4 (12.3)	58.0 (13.0)	60.8 (12.3)	58.6 (12.7)
Female	82 (33.9)	129 (23.8)	195 (29.1)	193 (28.5)	599 (28.1)
White	118 (48.8)	337 (62.3)	422 (63.1)	445 (65.7)	1322 (62.1)
TOPCAT, participants, No.	420	428	478	399	1725
Class I-II	181 (43.1)	259 (60.5)	342 (71.5)	340 (85.2)	1122 (65)
Class III-IV	239 (56.9)	169 (39.5)	136 (28.5)	59 (14.8)	603 (35)
Age, mean (SD), y	68.9 (10.1)	71.6 (9.5)	72.5 (9.3)	73.2 (9.4)	71.5 (9.7)
Female	233 (55.5)	239 (55.8)	242 (50.6)	147 (36.8)	861 (49.9)
White	302 (71.9)	335 (78.3)	388 (81.2)	330 (82.7)	1355 (78.6)

Abbreviations: HF-ACTION, Heart Failure: A Controlled Trial Investigating Outcomes of Exercise Training; KCCQ-os, the Kansas City Cardiomyopathy Questionnaire Overall Summary; KCCQINT, KCCQ Interpretability Study; NYHA, New York Heart Association; TOPCAT, Treatment of Preserved Cardiac Function Heart Failure with an Aldosterone Antagonist.

**Figure.**
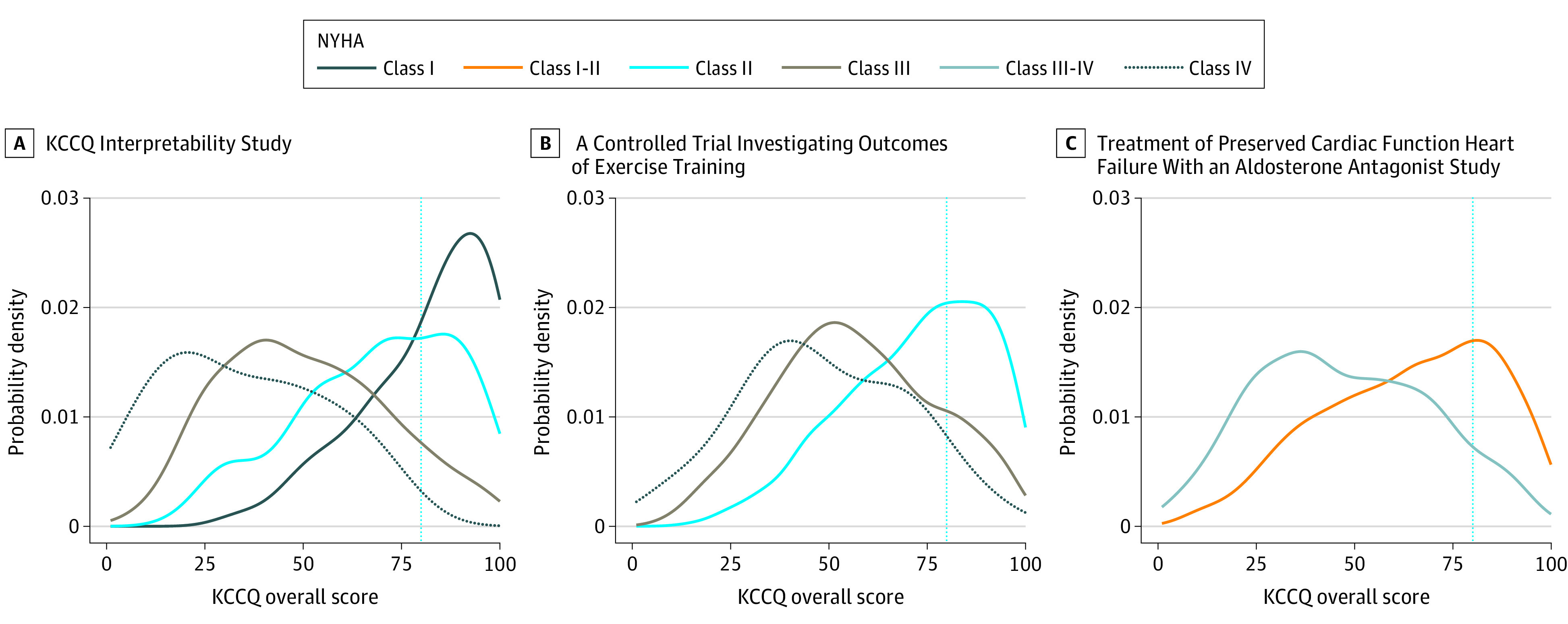
Distribution of New York Heart Association (NYHA) Classification by the Kansas City Cardiomyopathy Questionnaire Overall Summary (KCCQ-os) Scores by Clinical Studies Large variability is presented in the distribution of NYHA Classification by KCCQ-os scores within each clinical study. Blue dotted lines indicate KCCQ-os score of 80.

## Discussion

We found marked differences between patient-reported health status, as measured by the KCCQ-os, and clinician-assigned health status by NYHA class, with substantial overlap of KCCQ-os scores across each NYHA class and a substantial proportion of those with high KCCQ-os scores being classified as NYHA class II, III, and even IV. These findings have important implications for clinical trials seeking to enroll patients who are homogenously ill, including the potential for some with high KCCQ-os scores to not be able to improve (undermining power), some with low scores being too ill for potential treatment benefit, and great difficulty in translating the results to clinical practice. Given the known interphysician variability in assigning NYHA classes, better categorization of future patients might be achieved with the highly reproducible KCCQ. Some limitations of our study include the age of the studies (although the accuracy of NYHA is unlikely to have changed over time), limited generalizability of examining only 3 studies, and lack of adjustment for other patient factors, although this should not be necessary for 2 assessments of the same underlying construct.

The importance of patient health status has grown, as recently emphasized by the US Food and Drug Administration.^6^ Given the marked variability in patient reports of their health status across NYHA classes, future trials should consider patient-reported outcome measures as the basis for defining patient eligibility to enroll a more homogenous cohort of disease severity.

## References

[1] Bennett JA, Riegel B, Bittner V, Nichols J Validity and reliability of the NYHA classes for measuring research outcomes in patients with cardiac disease. Heart Lung. 2002;31(4):262-270. doi:10.1067/mhl.2002.124554 12122390

[2] Spertus JA, Jones PG Development and validation of a short version of the Kansas City Cardiomyopathy Questionnaire. Circ Cardiovasc Qual Outcomes. 2015;8(5):469-476. doi:10.1161/CIRCOUTCOMES.115.001958 26307129 PMC4885562

[3] Spertus J, Peterson E, Conard MW, ; Cardiovascular Outcomes Research Consortium Monitoring clinical changes in patients with heart failure: a comparison of methods. Am Heart J. 2005;150(4):707-715. doi:10.1016/j.ahj.2004.12.010 16209970

[4] O’Connor CM, Whellan DJ, Lee KL, ; HF-ACTION Investigators Efficacy and safety of exercise training in patients with chronic heart failure: HF-ACTION randomized controlled trial. JAMA. 2009;301(14):1439-1450. doi:10.1001/jama.2009.454 19351941 PMC2916661

[5] Pitt B, Pfeffer MA, Assmann SF, ; TOPCAT Investigators Spironolactone for heart failure with preserved ejection fraction. N Engl J Med. 2014;370(15):1383-1392. doi:10.1056/NEJMoa1313731 24716680

[6] US Food and Drug Administration Treatment for heart failure: endpoints for drug development guidance for industry. Updated June 2019 Accessed June 2020. https://www.fda.gov/regulatory-information/search-fda-guidance-documents/treatment-heart-failure-endpoints-drug-development-guidance-industry

